# Effectiveness of Kinesio taping on peripheral facial paralysis: A protocol for systematic review and meta-analysis

**DOI:** 10.1097/MD.0000000000023090

**Published:** 2020-11-13

**Authors:** Zai-hui Sun, Yan-ping Tian, Yan-fu Tan, Dan Tao, Wen-bo Li, Ji-lin Ding, Shuang-chun Ai

**Affiliations:** a School of Health Preservation and Rehabilitation, Chengdu University of Traditional Chinese Medicine; b Eye College of Chengdu University of Traditional Chinese Medicine; c Mianyang Hospital affiliated to Chengdu University of Traditional Chinese Medicine, Sichuan, China.

**Keywords:** Kinesio taping, meta-analysis, peripheral facial paralysis, protocol, systematic review

## Abstract

**Background:**

Peripheral facial paralysis is a rapid unilateral facial paralysis or paralysis of unknown etiology. Nearly 30% of patients leave sequela that have a negative impact on the patient's quality of life, both physically and psychologically. As its safety, convenience and effectiveness, Kinesio taping has been gradually used in the rehabilitation of peripheral facial paralysis. However, whether Kinesio taping is effective for peripheral facial paralysis is still unknown. The purpose of this systematic review (SR) and meta-analysis will summarize the current evidence of Kinesio taping used as an intervention for peripheral facial paralysis.

**Methods and analysis:**

We will search the following electronic databases for randomized controlled trials (RCTs) and controlled clinical trials (CCTs) to evaluate the effectiveness of Kinesio taping in treating peripheral facial paralysis: China National Knowledge Infrastructure (CNKI), Wanfang Date, SinoMed, Technology Periodical Database (VIP), PubMed, Embase, Web of Science, and The Cochrane Library. Each database will be searched from inception to April 2020. Studies that present clear descriptions of Kinesio taping in treating peripheral facial paralysis administration are published in peer-reviewed journals in any languages and are published in full will be taken into consideration. The entire process will include study selection, data extraction, risk of bias assessment and meta-analyses. Assessment of risk of bias and data synthesis will be conducted using Review Manager 5.3 software.

**Results:**

The current evidence on the Kinesio taping for managing peripheral facial paralysis will be illustrated using subjective reports and objective measures of performance. The primary outcome is the effective rate. Secondary outcomes include House-Brackmann scale, Portmann score, facial nerve conduction velocity, Facial Disability Index, Facial Disability Index include Facial Function score and social Function score.

**Conclusion:**

This protocol will present evidence on the efficacy of Kinesio taping in relieving peripheral facial paralysis.

**Ethics and dissemination:**

Since all the data used in this SR and meta-analysis have been published, ethical approval is not required for this review. The results of this SR will be published in a peer-reviewed journal or presented at conferences.

**INPLASY ID:**

(INPLASY2020100008).

## Abstract 2

Supplemental Digital Content is available in the text

## 1 Introduction

Peripheral facial paralysis (PFP), also known as idiopathic facial nerve paralysis, is the most common cranial nerve paralysis. Bell's palsy is the most common facial paralysis. PFP is a rapid unilateral facial paralysis or paralysis of unknown etiology. According to pathophysiological concept, it is by edema and facial nerve primary or secondary ischemia cause nerve compression and hypoxia. It will cause the facial muscles on the affected side to be partially or completely unable to move autonomously.^[1,2]^ Nearly 70% of patients with PFP recover completely, but 30% of patients leave sequela that have a negative impact on their quality of life, both physically and psychologically.^[3,4]^ The sequela of PFP include incomplete eye closure, crocodile tears, oral dysfunction during eating, dysphonia, muscle contractures, facial joint movements, and pain.^[4–6]^ Due to the inability to fully express emotions and facial aesthetics disorders will lead to the deprivation of social functions of patients.^[7]^ Active treatment and effective intervention measures should be taken clinically to improve clinical efficacy and reduce sequela.

There are many treatments for PFP, such as glucocorticoids and the use of antiviral drugs;^[8–10]^ Surgical treatment such as facial nerve decompression;^[11–13]^ traditional Chinese medicine treatments such as Chinese herbal decoction, acupuncture, moxibustion, etc.^[6,14–16]^ Physical therapy such as infrared polarized light irradiation and transcutaneous electrical stimulation.^[17,18]^ In recent years, physical therapy has been widely expanded in the treatment of PFP, Kinesio taping(KT) has also gradually used in the rehabilitation of PFP.^[19,20]^ KT was originally developed by Japanese scientist Dr. Kenso Kase in the 1970s. The physiological effects is to lift the skin, create extra space between the dermis and the muscles, reduce the pressure on the pain receptors located under the skin, thereby reducing pain. It also improves blood and lymph circulation, acting on “Gate-control of pain”, and affect the body system through “Neurofacilitation” (Stimulates the mechanoreceptors of the skin, causing positive changes to the nervous system).^[21,22]^

Although KT is increasingly used in the rehabilitation of PFP, its efficacy has not been fully proved. Up to now, there is no systematic review (SR) on the treatment of PFP with the use of KT. In this study, a comprehensive collection of clinical trials related to the treatment of PFP by KT were carried out to evaluate the effect of KT on PFP and the improvement of functions.

## 2 Materials and methods

The protocol for this systematic review was registered on INPLASY (INPLASY2020100008) and is available in full on the inplasy.com (https://doi.org/10.37766/inplasy2020.10.0008). This SR will be reported following the Preferred Reporting Items for Systematic Reviews and Meta-Analysis Protocol statement guidelines.^[23]^

## 3 Inclusion criteria for study selection

### 3.1 Type of studies

This review will include clinical randomized controlled trials (RCTs) and controlled clinical trials (CCTs) of KT for PFP patients without any language or publication status restrictions. Case reports, case series, crossover studies, laboratory studies, and uncontrolled trials will not be included.

### 3.2 Type of participants

Patients diagnosed with PFP (over 12 years old) will be included with no restriction on gender, race, or nation.

### 3.3 Type of interventions

Interventions will include any type of KT for improvement of symptoms of PFP. Studies combined with other interventions such as conventional medication, herbal medicines, acupuncture, moxibustion, physiotherapy will be considered for inclusion.

### 3.4 Type of comparators

The comparative interventions could be usual care, conventional rehabilitations, herbal medicines, acupuncture, moxibustion, or other active treatments.

### 3.5 Type of outcome measures

The primary outcome will be the total effective rate. Secondary outcomes will include House-Brackmann scale, Portmann score, facial nerve conduction velocity (NCV), Facial Disability Index (FDI), Facial Disability Index include Facial Function score (FDIp), and social Function score (FDIs).

## 4 Exclusion criteria for study selection

The exclusion criteria include:

1. Observational studies, case reports, cross-over trials, reviews
2. Central facial paralysis, Traumatic Facial Nerve Injury
3. Duplicated publications, requesting no results
4. Full text cannot be obtained
5. The original data is missing or incorrect, requesting no results
6. The study was divided into 3 groups or more
7. The treatment plan was not clear and the trial design was not rigorous

## 5 Data collection

### 5.1 Search strategy

We will search the following electronic databases for relevant trials from inception to present: China National Knowledge Infrastructure (CNKI), Wanfang Date, SinoMed, Technology Periodical Database (VIP), PubMed, Embase, Web of Science and The Cochrane Library. The search strategy was “subject terms+ free word”, there will be no language restrictions.

### 5.2 Studies selection

**Figure 1:**
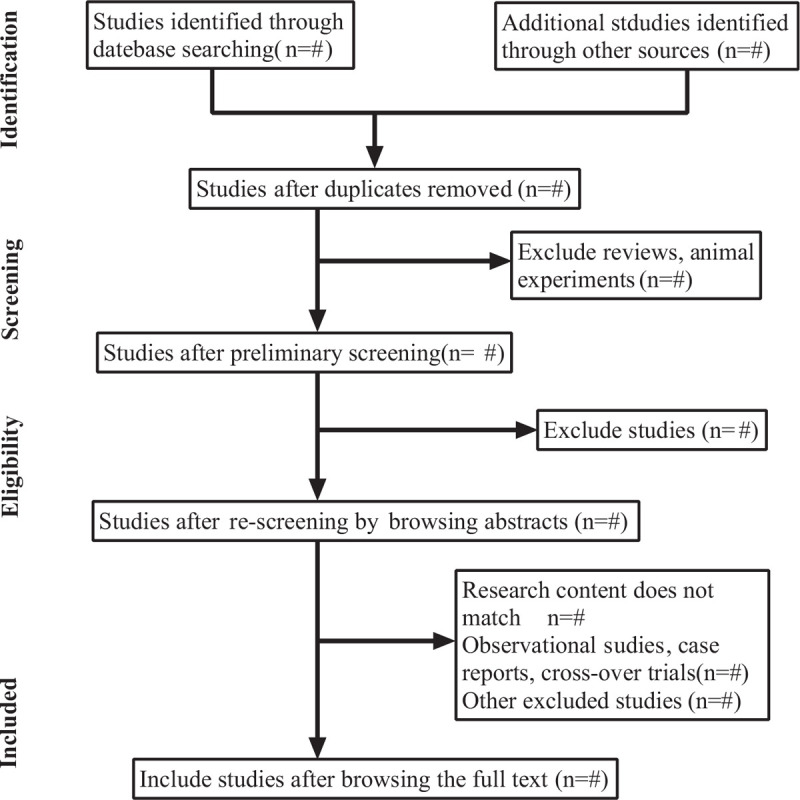
PRISMA flowchart.

We will use Endnote X9 to manage all the retrieved studies, and the duplicate studies will be filtered first. Two reviewers (ZHS and YPT) will independently screen the studies and extracted the data respectively according to the proposed inclusion criteria and exclusion criteria. In case of any disagreement, the 2 parties shall discuss and negotiate, and in case of any further disagreement, the third party expert (SCA) shall arbitrate whether to include the dispute or not. The study selection procedure will be performed in accordance with the Systematic Review and Meta-analysis (PRISMA) flowchart (see Fig. 1).

### 5.3 Data extraction and management

Two authors (ZHS and YPT) will read the full text and extract the following data according to the standard data collection form:

- General information: Publication year, first author, the title of the study;
- Study methods: study design, sample size, baseline comparability, randomization method, allocation concealment, blinding, integrity of result data, incomplete report or selecting report, other sources of bias;
- Participants: Inclusion and exclusion criteria;
- Intervention: Average age and age range of participants, treatment duration, and frequency;
- Control: Average age and age range of participants, type of control methods, treatment duration, and frequency;
- Outcomes: Outcome measures.

### 5.4 Risk of bias assessment

The risk of bias in included studies will be assessed independently by 2 reviewers (YFT and DT) using the Cochrane Handbook for Systematic Reviews of Interventions tool. A third reviewer (JLD) will mediate in situations of any disagreement. All judgments will be fully described, and the conclusions will be presented in the Risk of Bias figures and will be incorporated into the interpretation of review findings, by means of sensitivity analysis. The risk of bias domains includes the following: random sequence generation, allocation concealment, blinding, incomplete outcome data, selective reporting, and other bias. We will fully describe all the judgments, conclusions will be presented in the Risk of Bias figures, and incorporate interpretations of the review results through sensitivity analysis. The risk of bias of each domain will be judged as “unclear”, “low risk” or “high risk”.^[24]^

## 6 Data analysis and methods

### 6.1 Dealing with missing data

If the primary results are lacking, incomplete, or unclear, we will contact the original authors for the missing data via email. If the missing data cannot be obtained from the original authors we will analyze the available data and just do a narrative analysis.

### 6.2 Data analysis

We will use RevMan 5.3 software provided by the Cochrane collaboration to process the meta-analysis. The relative risk will be used to analyze dichotomous outcomes, while the mean difference or standardized mean difference will be used to analyze continuous outcomes. We will measure heterogeneity in each of the included research questions by using the χ^2^ test. Fixed-effects model will be used if there is homogeneity between the studies (*P* > .1, *I*^2^ ≤ 50%). If there is obvious heterogeneity among the studies (*P* ≤ .1, *I*^2^ > 50%), we will first find the 1 or more outlier studies that causes of heterogeneity through 3 methods: subgroup analysis, sensitivity analysis, and meta-regression analyses. And conduct subgroup research or delete the research that leads to heterogeneity, and then use the fixed-effects model to merge the effect size for meta-analysis. If the reason for the heterogeneity cannot be found, the random-effects model can be used in the acceptable range (*I*^2^ < 75%). If the heterogeneity is too large (*I*^2^ ≥ 75%), then no merger will be carried out and only a descriptive analysis will be done.

### 6.3 Subgroup analysis

If possible, we will conduct subgroup analyses based on age, sex, treatment duration, treatment frequency, and basic treatment (e.g., conventional medication, herbal medicines, acupuncture, moxibustion, physiotherapy).

### 6.4 Sensitivity analysis

The results of one or more outlier studies will conflict with other studies and may become a source of heterogeneity. In order to ensure the quality of meta-analysis we will perform a sensitivity analysis to exclude outliers.

### 6.5 Publication bias

Publication bias will be assessed graphically using funnel plots if a meta-analysis includes 10 or more studies. If funnel plots are asymmetric, we will try to interpret funnel plot asymmetry.

### 6.6 Ethics and dissemination

The data used in this SR will be collected based on published studies. Based on this, no ethical approval is required. According to the PRISMA guidelines, we will publish the results of this SR in a peer-reviewed scientific journals.

## 7 Discussion

PFP is a kind of unilateral facial nerve paresis or paralysis of unknown cause. The etiology of PFP include cold irritation, viral infection, etc., causing inflammation, and edema in the styloid mastoid foramen, resulting in facial nerve compression and ischemia, causing facial nerve paralysis.^[1,25]^ PFP is easy to diagnose clinically. After treatment, most patients can recover completely without affecting the survival rate and life expectancy, and usually the prognosis is good. However, patients will experience greater mental stress before they get better, and even with proper treatment, up to 30% of patients develop long-term sequela, such as permanent facial paralysis, stiffness, contracture, and facial asymmetry. Therefore, the time to complete recovery and the effect of treatment are of great concern to patients.^[26]^ With the introduction of KT, there is an effective method for the rehabilitation of PFP, its mechanism of action is as follows:

When the patient is attached to the KT, there will be obvious muscle tension and tightness. The current situation of facial muscle weakness and facial numbness will also be alleviated, which is of great help to relieve patients psychological pressure and improve patients confidence in treatment. At the same time, KT can stimulate the skin mechanoreceptors, increase sensory input, and proprioceptive feedback.^[27–29]^

Because of its elastic effect, KT lifting the skin, increases skin folds, reduces the pressure in the surrounding tissues of the nerves, thereby increasing blood circulation and lymph flow, which can promote the absorption of edema and the diffusion and metabolism of inflammatory factors, this creates a good internal environment for nerve recovery.^[29–32]^

KT can assist muscle contraction. If the direction of KTs tension matches the direction of muscle contraction, the recoil force of the KT can be transmitted to the fascia. This effect increases the excitability of the motor unit and induces muscle spindle reflex. This strengthens the weak muscles help to realign structures around the face and modulate muscle normal activities.^[33,34]^

However, there is still lack of valid evidence to support that KT is effective for PFP. Therefore, the purpose of this meta-analysis is mainly to evaluate the effectiveness and safety of KT for the treatment of PFP. Provide reliable evidence for its wide application. Search strategy of Embase.

## Author contributions

**Data curation:** Yan-ping Tian.

**Methodology:** Ji-lin Ding, Shuang-chun Ai.

**Resources:** Yan-fu Tan, Dan Tao.

**Supervision:** Wen-bo Li.

**Writing – original draft:** Zai-hui Sun.

**Writing – review & editing:** Shuang-chun Ai.

## Supplementary Material

Supplemental Digital Content

## References

[1] Baugh RF Basura GJ Ishii LE Clinical Practice Guideline: Bell's palsy. Otolaryngol Head Neck Surg 2013;149:S1–27. 10.1177/0194599813505967 24189771

[2] Mahesh SG Nayak DR Balakrishnan R Modified Stennert's protocol in treating acute peripheral facial nerve paralysis: our experience. Indian J Otolaryngol Head Neck Surg 2013;65:214–8. 24427569 10.1007/s12070-011-0440-2 PMC3696149

[3] Baek S Kim YH Kwon Y The utility of facial nerve ultrasonography in Bell's palsy. Otolaryngol Head Neck Surg 2020;162:186–92. 31870206 10.1177/0194599819896298

[4] Bylund N Hultcrantz M Jonsson L Quality of life in Bell's palsy: correlation with Sunnybrook and House-Brackmann over time. Laryngoscope 2020;doi: 10.1002/lary.28751. 10.1002/lary.28751 32463963

[5] George E Richie MB Glastonbury CM Facial nerve palsy: clinical practice and cognitive errors. Am J Med 2020;133:1039–44. 32445717 10.1016/j.amjmed.2020.04.023

[6] Dan-dan J Jing Y Meng G Efficacy of acupuncture-moxibustion on peripheral facial paralysis at different time points: a meta-analysis. Chin Acupunct Moxibust 2020;40:664–8. 10.13703/j.0255-2930.20190721-k0003 32538021

[7] Guang-hui D Wen-jing M Bin L Factors related to outcome of idiopathic facial palsy. Chin J Rehab Theory Pract 2016;22:464–8.

[8] Urban E Volk GF Geissler K Prognostic factors for the outcome of Bells’ palsy: a cohort register-based study. Clin Otolaryngol 2020;45:754–61. 10.1111/coa.13571 32395899

[9] Linghao M Manying G Baoxing Y Clinical observation of different administration methods of glucocorticoid in treating Bell's palsy. Chin J Pract Nerv Dis 2020;23:1005–8.

[10] Wang W Jiang R Liu N Electroacupuncture is effective for peripheral facial paralysis: a meta-analysis. Evid-Based Compl Alt 2020;2020:1–1. 10.1155/2020/5419407 PMC7150689 32328134

[11] Lee S Seong J Kim YH Clinical implication of facial nerve decompression in complete Bell's palsy: a systematic review and meta-analysis. Clin Exp Otorhinolar 2019;12:348–59. 10.21053/ceo.2019.00535 PMC6787481 31487771

[12] Kim J Facial nerve decompression for Bell's palsy: an endless debate. Clin Exp Otorhinolar 2019;12:331–2. 10.21053/ceo.2019.01515 PMC6787471 31575104

[13] Zhiying N Yitao M Anquan P Facial nerve decompression for peripheral facial paralysis. Chin J Otol 2014;415–8.

[14] Li D Li J Ye X Early treatment of suspension moxibustion for Bell's palsy in acute stage. Chin Acupunct Moxibust 2020;40:123. 10.13703/j.0255-2930.20190101-k00014 32100495

[15] Wenting L Zewei C Zhiyong W Clinical effect and safety of Buhuangsiwu Decoction in treating patients with peripheral facial paralysis. J Jinan Univ 2018;39:332–8.

[16] Bing W Jinhong Y Feng C Evaluation of therapeutic effect of different Chinese medicine treatments on peripheral facial paralysis. J Tradit Chin Med 2017;58:1929–33.

[17] Mäkelä E Venesvirta H Ilves M Facial muscle reanimation by transcutaneous electrical stimulation for peripheral facial nerve palsy. J Med Eng Technol 2019;43:155–64. 31305190 10.1080/03091902.2019.1637470

[18] Pan-pan L Ming-zhu H 154 cases of peripheral facial paralysis by physical assisted therapy. J Nongken Med 2019;41:46–8.

[19] Alptekin DO Acupuncture and Kinesio Taping for the acute management of Bell's palsy: a case report. Complement Ther Med 2017;35:1–5. 29154053 10.1016/j.ctim.2017.08.013

[20] Fei Q Haixia Y Yanwei L Clinical observation of peripheral facial neuritis treated with acupuncture combined with Kinesio Taping. Chin J Rehab Med 2017;32:424–7. 450.

[21] Melese H Alamer A Hailu Temesgen M Effectiveness of Kinesio taping on the management of knee osteoarthritis: a systematic review of randomized controlled trials. J Pain Res 2020;13:1267–76. 32547187 10.2147/JPR.S249567 PMC7266391

[22] Abolhasani M Halabchi F Afsharnia E Effects of kinesiotaping on knee osteoarthritis: a literature review. J Exerc Rehabil 2019;15:498–503. 31523668 10.12965/jer.1938364.182 PMC6732535

[23] Shamseer L Moher D Clarke M Preferred reporting items for systematic review and meta-analysis protocols (PRISMA-P) 2015: elaboration and explanation. BMJ 2015;349:g7647. 10.1136/bmj.g7647 25555855

[24] Sterne JAC Savović J Page MJ RoB 2: a revised tool for assessing risk of bias in randomised trials. BMJ 2019;l4898. 31462531 10.1136/bmj.l4898

[25] Zhang W Xu L Luo T The etiology of Bell's palsy: a review. J Neurol 2020;267:1896–905. 30923934 10.1007/s00415-019-09282-4 PMC7320932

[26] Yoo MC Soh Y Chon J Evaluation of factors associated with favorable outcomes in adults with Bell palsy. JAMA Otolaryngol Head Neck Surg 2020;146:256. 31971554 10.1001/jamaoto.2019.4312 PMC6990801

[27] Hassan BS Abbass ME Elshennawy S Systematic review of the effectiveness of Kinesio taping for children with brachial plexus injury. Physiother Res Int 2019;25. 10.1002/pri.1794 31231910

[28] Desjardins-Charbonneau A Roy JS Dionne CE The efficacy of taping for rotator cuff tendinopathy: a systematic review and meta-analysis. Int J Sports Phys Ther 2015;10:420–33. 26346114 PMC4527190

[29] Hui S Xin X Effect of Kinesio taping on rehabilitation and prevention for sport injuries (review). Chin J Rehab Theory Pract 2019;25:64–9.

[30] Williams S Whatman C Hume PA Kinesio taping in treatment and prevention of sports injuries. Sports Med 2012;42:153–64. 22124445 10.2165/11594960-000000000-00000

[31] Bin F Lin L Huan Z Effect of Kinesio taping on pain in myofascial pain syndrome: a meta-analysis. Chin J Rehab Theory Pract 2018;24:347–52.

[32] Jiang Z Yongkang H Du Shuang Efficacy of kinesio taping on post stroke shoulder pain: a meta-analysis. Chin J Evid-Based Med 2019;19:673–9.

[33] Li Y Yin Y Jia G Effects of kinesiotape on pain and disability in individuals with chronic low back pain: a systematic review and meta-analysis of randomized controlled trials. Clin Rehabil 2019;33:596–606. 30526011 10.1177/0269215518817804

[34] Guo-hai Z Ren-wei W Progress and prospect in research about Kinesio taping on human performance and the related mechanis. China Sport Sci Technol 2015;51:73–80.

