# Supplementary figures and images for: Effectiveness of Kinesio taping on peripheral facial paralysis: A protocol for systematic review and meta-analysis
Source: Medicine (Baltimore). 2020 Nov 13;99(46):e23090. doi: 10.1097/MD.0000000000023090 (PMC7668467; doi:10.1097/MD.0000000000023090)

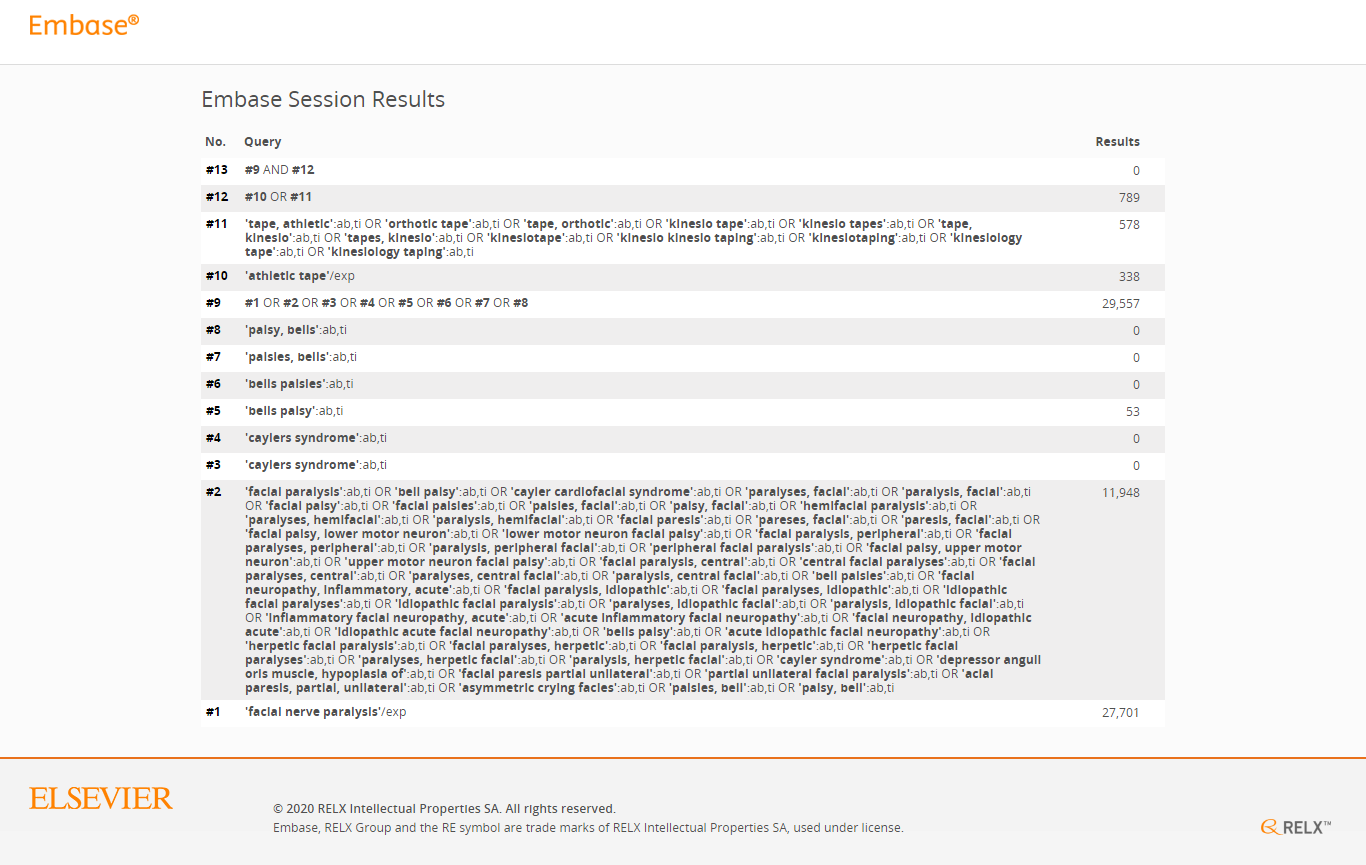


**Search strategy of Embase**

Supplement: Supplemental Digital Content [file medi-99-e23090-s001.docx]
